# Low-flow time and neurological outcomes in AMI patients undergoing ECPR: a retrospective cohort study

**DOI:** 10.3389/fcvm.2026.1942551

**Published:** 2026-09-14

**Authors:** Bingwei Liang, Qian Yu, Lijun Xu, Yanwei Cheng

**Affiliations:** Department of Emergency Medicine, Henan Provincial People’s Hospital, Zhengzhou, China

**Keywords:** acute myocardial infarction, cardiac arrest, cerebral performance category, extracorporeal cardiopulmonary resuscitation (ECPR), low-flow time, neurological outcome, no-flow time, retrospective cohort study

## Abstract

**Background:**

Acute myocardial infarction (AMI) complicated by refractory cardiac arrest (CA) carries extremely high mortality and neurological disability rates. Extracorporeal cardiopulmonary resuscitation (ECPR) is a rescue intervention for this population, but outcomes after its use vary widely across individuals. Low-flow time (the interval from initiation of chest compressions to establishment of stable ECMO flow) is an important prognostic factor among ECPR-treated patients. Its association with outcomes in Chinese patients with AMI-related CA remains incompletely characterized.

**Methods:**

We conducted a retrospective cohort study of 161 eligible adult patients with AMI-related refractory CA who received ECPR at our tertiary center between January 2019 and December 2025. The primary endpoint was 30-day unfavorable neurological outcome [Cerebral Performance Category (CPC) score 3-5]. Multivariable logistic regression evaluated low-flow time continuously as the primary exposure. RCS regression assessed possible nonlinearity, whereas ROC analysis provided a secondary, exploratory cohort-specific classification. We performed an exact-timing sensitivity analysis and a full-cohort interaction analysis to assess heterogeneity by arrest setting.

**Results:**

Among the 161 enrolled patients, 118 (73.3%) had 30-day unfavorable neurological outcomes, including 104 deaths (64.6%). After multivariable adjustment, each additional minute of low-flow time was associated with higher odds of unfavorable outcome (aOR 1.065, 95% CI 1.036–1.096; *P* < 0.001). The association remained in 132 patients with exact timing (aOR 1.082, 95% CI 1.042–1.124; *P* < 0.001). In a parsimoniously adjusted interaction model, the estimated aOR per minute was 1.061 (95% CI 1.027–1.096) for IHCA and 1.072 (95% CI 1.023–1.124) for OHCA; the interaction was not statistically detectable (*P* = 0.708). RCS did not detect nonlinearity (*P* = 0.660). The exploratory 50.9-min classification was internally derived and its bootstrap cutpoints were widely distributed.

**Conclusions:**

Among selected AMI patients who received ECPR, increasing low-flow time was progressively associated with unfavorable 30-day neurological outcome, with no detected nonlinear inflection or evidence of effect modification by arrest setting. These findings are prognostic among ECPR-treated patients and do not define when ECPR should or should not be initiated. The internally derived 50.9-min classification is exploratory and is not an eligibility threshold.

## Introduction

Acute myocardial infarction (AMI) complicated by refractory cardiac arrest (CA) is among the most difficult scenarios in extracorporeal cardiopulmonary resuscitation (ECPR). Even with standardized cardiopulmonary resuscitation (CPR) and timely reperfusion, conventional CPR (CCPR) provides only a fraction of normal cardiac output during refractory arrest, which may be insufficient to sustain vital organ perfusion (1). Prolonged CCPR not only worsens systemic ischemic-hypoxic injury but also reduces the likelihood of return of spontaneous circulation and accelerates irreversible multi-organ injury.

ECPR is used as a rescue strategy for this otherwise frequently fatal scenario. By rapidly establishing stable extracorporeal perfusion, it creates a window for definitive interventions such as emergency percutaneous coronary intervention (PCI). Randomized and observational evidence of benefit remains heterogeneous (2–5), and outcomes vary considerably across individuals, depending on age, comorbidities, arrest circumstances, CPR quality, and the timing of extracorporeal membrane oxygenation (ECMO) initiation.

Among these variables, low-flow time—defined here as the interval from initiation of chest compressions to stable ECMO flow—is considered an important prognostic factor. Longer low-flow times have consistently been linked to worse neurological outcomes after ECPR (6–9). In AMI patients who suffer CA, pre-existing myocardial ischemia may be compounded by extended low-flow states; delayed coronary reperfusion may further aggravate myocardial and cerebral injury (10). No-flow time, defined as the interval from collapse to the first chest compression, may provide additional prognostic information, particularly in out-of-hospital cardiac arrest (11).

Despite this well-recognized relationship, several gaps remain. Most published studies combine heterogeneous cardiac-arrest etiologies and do not focus specifically on AMI-CA, in which coronary occlusion, emergency reperfusion, and low-flow ischemia coexist. Arrest setting and timing precision may also alter the interpretation of low-flow time. Reported data-derived cutpoints vary widely and should not be conflated with biological inflection points or ECPR eligibility criteria (12, 13). Detailed AMI, coronary-treatment, no-flow, arrest-setting, and timing-quality data from Chinese ECPR cohorts remain limited.

To address these gaps, we conducted a retrospective analysis of 161 AMI patients with refractory CA who received ECPR at our 3,900-bed tertiary center between January 2019 and December 2025. The primary purpose was to characterize the continuous prognostic association between low-flow time and outcome among patients already treated with ECPR. Secondary aims were to assess nonlinearity, evaluate timing-record sensitivity and arrest-setting heterogeneity, and describe an exploratory cohort-specific discriminator. The study was not designed to compare ECPR with non-ECPR care or to establish an ECPR initiation or exclusion threshold.

## Methods

### Study design and population

We performed a retrospective cohort study at a 3,900-bed tertiary care center in China. Eligible adult patients (≥18 years) with AMI-related refractory CA receiving emergency ECPR between January 2019 and December 2025 were screened. Of 203 patients screened, 42 were excluded: 12 did not meet the AMI criteria, 8 had pre-existing severe neurological disease, 10 had missing key data, 6 had incomplete pre-transfer records, and 6 additional cases identified during source-data re-verification had serious contradictions in recorded event times that prevented reliable determination of low-flow time. The final cohort comprised 161 patients. Refractory CA was operationally defined as persistent absence of sustained return of spontaneous circulation despite conventional CPR and advanced life support, prompting activation of the institutional ECPR pathway. A uniform minimum duration of advanced life support before ECPR activation could not be verified retrospectively.

Inclusion criteria were: (1) confirmed AMI (STEMI or NSTEMI) with refractory in-hospital or out-of-hospital CA; (2) ECPR initiated as rescue therapy after failed conventional CPR; (3) complete data on low-flow time and 30-day neurological status; and (4) age ≥18 years. Exclusion criteria were: (1) CA from non-AMI causes; (2) pre-existing severe neurological deficit or end-stage organ failure; (3) ECMO placed for indications other than resuscitation; (4) missing low-flow time or 30-day CPC data; (5) incomplete pre-transfer resuscitation records that prevented confirmation of the primary timing variable; and (6) serious contradictions in recorded event times that prevented reliable determination of low-flow time.

The national multicenter project “Survey of the Incidence, Mortality, and Risk Factors of Cardiac Arrest in the Chinese Population” (task no. 2018FY10060005), in which Henan Provincial People's Hospital participated as a Central China regional site, received ethics approval from the Ethics Committee of Qilu Hospital, Shandong University (approval no. Lunshen-2019012). The present retrospective analysis used de-identified clinical records generated at Henan Provincial People's Hospital. The Ethics Committee of Henan Provincial People's Hospital reviewed the manuscript and related study materials and confirmed that written informed consent from the participants or their legal representatives could be waived because the study involved no additional examinations, clinical interventions, or patient contact and posed no more than minimal risk.

Reporting guideline: This retrospective cohort study is reported in accordance with the Strengthening the Reporting of Observational Studies in Epidemiology (STROBE) statement. The completed STROBE checklist for cohort studies is provided in the Supplementary Material.

### AMI and coronary assessment

Two independent investigators extracted clinical data from electronic health records, the cardiac catheterization database, and the institutional ECMO registry. Discrepancies were resolved by consensus with a senior cardiologist specializing in ECPR and AMI. AMI was identified from contemporaneous STEMI or NSTEMI diagnoses made by the treating cardiology team. The analytic dataset retained AMI subtype, peak troponin I, receipt of PCI, culprit vessel, post-PCI Thrombolysis in Myocardial Infarction (TIMI) flow grade, and the recorded reason for no PCI. The source records did not permit retrospective reconstruction of every diagnostic component, and a uniformly analyzable symptom-onset time was unavailable.

Culprit vessel and post-PCI TIMI grade were summarized only among the 130 patients receiving PCI, and reasons for no PCI were summarized only among the 31 patients not receiving PCI. Patient-level angiography and reperfusion intervals were not analyzed because 68 of 130 PCI records did not satisfy the temporal consistency rule used for acute interval analysis (a negative interval, angiography more than 24 h after the recorded arrest, or reperfusion preceding angiography); no patient-level correction was inferred. Echocardiographic measurements were not compared because their timing relative to arrest, ECPR initiation, and subsequent support was not standardized. Swan-Ganz measurements were not available as a sufficiently complete, time-anchored series.

### Cardiac arrest and ECPR variables

Collected variables included demographics (age, sex), comorbidities (hypertension, diabetes, smoking, known coronary artery disease), AMI characteristics (STEMI vs. NSTEMI, peak troponin I, receipt of PCI), and resuscitation details (arrest location, witnessed status, CPR duration, and initial rhythm). Additional physiological severity was captured by the APACHE II score and admission lactate level. No-flow time was defined as the interval from documented collapse or arrest to initiation of the first chest compression. Low-flow time was defined as the interval from initiation of chest compressions to establishment of stable ECMO flow. Arrest and CPR timestamps were retrieved from nursing, resuscitation, prehospital emergency, or family records. The precision of the arrest/CPR timing records was classified as exact, estimated, or indeterminate. Bystander CPR was evaluated only among OHCA patients. Detailed operational definitions and analysis handling are provided in Supplementary Table S1.

All patients were managed by the institutional ECPR team using venoarterial ECMO as the default mode; venoarterial-venous configuration was used when clinically indicated. Separately recorded CPR-duration and ECMO-startup fields had different operational time anchors and were reported descriptively but were not substituted for the primary low-flow definition. Reliable patient-level Impella and intra-aortic balloon pump use could not be retrieved for the full cohort.

All patients were managed under a center-level temperature-control protocol aimed at normothermia and fever prevention; surface cooling was the predominant method. Induced hypothermic temperature control at 32 °C–34 °C was not used. Individual target temperatures, device details, treatment durations, and rewarming rates were not captured as analyzable fields; temperature management was therefore described at the protocol level and was not entered into regression models (14).

### Outcome definition

The primary endpoint was unfavorable neurological outcome at 30 days, defined as CPC 3-5; CPC 1-2 represented favorable outcome. Death was coded as CPC 5. The complete CPC distribution and 30-day mortality were reported so that death could be distinguished from survival with severe neurological disability. Stored favorable/unfavorable indicators were cross-checked against the recorded 30-day CPC score. GOS and a qualitative neurological-prognosis field were not used because their categorization was not concordant with the prespecified CPC endpoint.

### Statistical analysis

Baseline characteristics were compared between patients with favorable and unfavorable 30-day neurological outcomes. Normally distributed continuous data were expressed as mean ± standard deviation and analyzed with Welch's *t* test, while non-normal continuous variables were presented as median (interquartile range) and compared using the Mann–Whitney *U* test. Categorical variables were summarized as counts and proportions, with between-group differences assessed by the chi-square test or Fisher's exact test. All tests were two-sided. *P* values for baseline and univariable comparisons were descriptive, and no adjustment was made for multiple comparisons.

Candidate covariates were selected on clinical grounds and data availability, informed by prior ECPR literature. The primary multivariable model included low-flow time, age, sex, diabetes mellitus, prior coronary artery disease, witnessed arrest, lactate, and initial shockable rhythm; it was not prespecified before data collection. The smaller outcome group comprised 43 patients, corresponding to 5.4 patients per predictor parameter. To limit overfitting, newly retrieved covariates were not entered together. Internal validation used 1,000 bootstrap samples to estimate optimism. The exact-timing sensitivity analysis repeated the primary model among patients classified as exact and also used a parsimonious model including age, witnessed arrest, low-flow time, lactate, and initial shockable rhythm. Arrest-setting heterogeneity was evaluated in the full cohort with an interaction between mean-centered continuous low-flow time and OHCA; the parsimonious interaction model adjusted for age, witnessed arrest, lactate, and initial shockable rhythm.

For missing data management, we first evaluated the completeness of all variables. The primary outcome and all primary-model variables were complete for the 161 analyzed patients, so no imputation was performed. Conditionally applicable coronary and OHCA variables were analyzed with their stated denominators (Supplementary Table S2). No *a priori* sample-size calculation was performed; the study size was determined by eligible cases available during the study period.

Restricted cubic spline (RCS) terms with three knots at the 10th, 50th, and 90th percentiles assessed departure from linearity. The continuous model and RCS analysis were primary for interpretation. ROC analysis of low-flow time alone and its maximum-Youden classification were secondary and exploratory; the 50.85-min value was operationalized as <50.9 versus ≥50.9 min only to describe within-cohort discrimination, not a biological change point or ECPR eligibility threshold. The AUC and cutpoint distribution were assessed using 2,000 bootstrap samples. Model discrimination was summarized by the C-statistic; calibration by the Hosmer-Lemeshow test; multicollinearity by variance-inflation factors; and model optimism by 1,000 bootstrap samples. Statistical analyses were performed using R version 4.6.1, and figures were prepared using GraphPad Prism version 10.

## Results

### Cohort and baseline characteristics

Of 203 patients screened, 42 were excluded: 12 did not meet the AMI criteria, 8 had pre-existing severe neurological disease, 10 had missing key data, 6 had incomplete pre-transfer records, and 6 additional cases identified during source-data re-verification had serious contradictions in recorded event times that prevented reliable determination of low-flow time. The final cohort comprised 161 patients. Overall, 43 (26.7%) patients had a favorable 30-day neurological outcome (CPC 1-2), and 118 (73.3%) had unfavorable neurological outcomes (CPC 3-5). Table 1 compares baseline and clinical characteristics by neurological status. In unadjusted comparisons, patients with unfavorable outcomes were older and more frequently had prior coronary artery disease, unwitnessed arrest, and a nonshockable initial rhythm; they also had longer low-flow time, higher APACHE II score, higher lactate, and a longer recorded ECMO-startup interval (all *P* < 0.05).

**Table 1:** Baseline, arrest, AMI, and ECPR characteristics by 30-day CPC outcome.

Variable	Overall (*n* = 161)	CPC 1-2 (*n* = 43)	CPC 3-5 (*n* = 118)	*P* value
Age (years), mean ± SD	60.58 ± 13.41	55.35 ± 12.98	62.49 ± 13.11	0.003
Male sex, *n*/*N* (%)	115/161 (71.4%)	26/43 (60.5%)	89/118 (75.4%)	0.063
Hypertension, *n*/*N* (%)	100/161 (62.1%)	23/43 (53.5%)	77/118 (65.3%)	0.173
Diabetes mellitus, *n*/*N* (%)	38/161 (23.6%)	14/43 (32.6%)	24/118 (20.3%)	0.106
Smoking history, *n*/*N* (%)	48/161 (29.8%)	12/43 (27.9%)	36/118 (30.5%)	0.750
Coronary artery disease history, *n*/*N* (%)	82/161 (50.9%)	16/43 (37.2%)	66/118 (55.9%)	0.036
STEMI, *n*/*N* (%)	121/161 (75.2%)	30/43 (69.8%)	91/118 (77.1%)	0.340
Peak troponin I (ng/mL), median (IQR)	28.37 (17.98–39.46)	31.36 (21.16–40.06)	27.48 (17.63–38.20)	0.326
PCI treatment, *n*/*N* (%)	130/161 (80.7%)	37/43 (86.0%)	93/118 (78.8%)	0.303
LAD culprit vessel (among PCI recipients), *n*/*N* (%)	47/130 (36.2%)	15/37 (40.5%)	32/93 (34.4%)	0.511
Post-PCI TIMI grade 3 flow (among PCI recipients), *n*/*N* (%)	109/130 (83.8%)	28/37 (75.7%)	81/93 (87.1%)	0.110
In-hospital cardiac arrest, *n*/*N* (%)	110/161 (68.3%)	29/43 (67.4%)	81/118 (68.6%)	0.885
Witnessed arrest, *n*/*N* (%)	107/161 (66.5%)	35/43 (81.4%)	72/118 (61.0%)	0.015
Bystander CPR (among OHCA), *n*/*N* (%)	32/51 (62.7%)	12/14 (85.7%)	20/37 (54.1%)	0.037
No-flow time (minutes), median (IQR)	0.80 (0.60–2.70)	0.80 (0.60–2.85)	0.80 (0.60–2.65)	0.922
Recorded CPR duration (minutes), median (IQR)	11.50 (7.20–16.80)	13.20 (7.65–17.60)	10.85 (7.22–16.55)	0.347
Low-flow time (minutes), median (IQR)	55.50 (39.90–65.60)	35.50 (24.95–55.80)	58.50 (50.55–70.05)	<0.001
Initial shockable rhythm, *n*/*N* (%)	64/161 (39.8%)	24/43 (55.8%)	40/118 (33.9%)	0.012
Arrest/CPR timing classified as exact, *n*/*N* (%)	132/161 (82.0%)	32/43 (74.4%)	100/118 (84.7%)	0.131
VAV ECMO mode, *n*/*N* (%)	12/161 (7.5%)	1/43 (2.3%)	11/118 (9.3%)	0.184
Recorded ECMO-startup interval (minutes), median (IQR)	44.50 (31.60–58.80)	32.10 (22.10–43.00)	50.50 (36.00–62.65)	<0.001
APACHE II score, median (IQR)	28.00 (23.00–35.00)	27.00 (20.00–31.00)	29.00 (23.00–35.75)	0.032
Lactate (mmol/L), median (IQR)	8.71 (6.05–12.00)	7.39 (5.14–9.67)	9.00 (6.19–12.00)	0.028

Values are *n*/*N* (%), mean ± SD, or median (IQR). Bystander CPR uses OHCA patients as the denominator. Culprit-vessel and post-PCI TIMI rows use PCI recipients as the denominator. *P* values are descriptive and were not used to select primary-model covariates. AMI, acute myocardial infarction; CPC, cerebral performance category; CPR, cardiopulmonary resuscitation; ECMO, extracorporeal membrane oxygenation; IHCA, in-hospital cardiac arrest; OHCA, out-of-hospital cardiac arrest; PCI, percutaneous coronary intervention; TIMI, thrombolysis in myocardial infarction.

The final cohort included 110 patients (68.3%) with in-hospital cardiac arrest (IHCA) and 51 (31.7%) with OHCA. Median no-flow time was 0.8 min (IQR 0.6–2.7); it was 0.7 min (IQR 0.5–0.8) for IHCA and 3.6 min (IQR 2.8–4.4) for OHCA. All IHCA no-flow intervals were ≤1 min and all cohort values were ≤5 min. Median low-flow time was 55.5 min (IQR 39.9–65.6).

Among 130 PCI recipients, the culprit vessel was LAD in 47, LCX in 33, RCA in 38, LAD + RCA in 7, and LAD + LCX + RCA in 5; post-PCI TIMI grade 3 flow was recorded in 109/130 (83.8%). Among 31 patients not receiving PCI, recorded reasons were family refusal (*n* = 11), active bleeding or severe coagulopathy (*n* = 10), mild angiographic disease not requiring PCI (*n* = 5), diffuse multivessel disease favoring surgery (*n* = 3), and inability to tolerate intervention because of severe instability (*n* = 2).

### Thirty-day outcomes

At 30 days, 43/161 (26.7%) had CPC 1-2 and 118/161 (73.3%) had CPC 3-5. The CPC distribution was CPC 1, 28 (17.4%); CPC 2, 15 (9.3%); CPC 3, 7 (4.3%); CPC 4, 7 (4.3%); and CPC 5, 104 (64.6%) (Table 2). Thirty-day mortality was 104/161 (64.6%), while 14 surviving patients had CPC 3-4.

**Table 2:** Distribution of 30-day cerebral performance category.

Category	Description	*n* (%)
CPC 1	Good cerebral performance	28 (17.4%)
CPC 2	Moderate cerebral disability	15 (9.3%)
CPC 3	Severe cerebral disability	7 (4.3%)
CPC 4	Coma or vegetative state	7 (4.3%)
CPC 5	Death	104 (64.6%)
CPC 1-2	Favorable neurological outcome	43 (26.7%)
CPC 3-5	Unfavorable neurological outcome	118 (73.3%)

CPC, cerebral performance category. CPC 1-2 indicates favorable neurological outcome; CPC 3-5 indicates unfavorable neurological outcome. Percentages are based on the total cohort of 161 patients.

### Univariable and multivariable logistic regression

Univariable analysis (Table 3) identified older age, prior coronary artery disease, unwitnessed arrest, nonshockable rhythm, longer low-flow time, higher APACHE II score, higher lactate, and a longer recorded ECMO-startup interval as associated with CPC 3-5 at conventional significance levels. No statistically detectable association was observed between no-flow time and outcome, but its range was narrow and strongly dependent on arrest location. Primary-model covariates were selected on clinical grounds rather than by univariable *P* values.

**Table 3:** Univariable logistic regression analysis of factors associated with unfavorable neurological outcome in AMI patients undergoing ECPR.

Variable	OR (95% CI)	*P* value	*n*
Age, per year	1.04 (1.01–1.07)	0.004	161
Male sex	2.01 (0.96–4.21)	0.066	161
Hypertension	1.63 (0.80–3.32)	0.175	161
Diabetes mellitus	0.53 (0.24–1.15)	0.109	161
Smoking history	1.13 (0.52–2.46)	0.750	161
Coronary artery disease history	2.14 (1.05–4.39)	0.037	161
STEMI	1.46 (0.67–3.19)	0.341	161
Peak troponin I, per 10 ng/mL	0.91 (0.72–1.15)	0.449	161
PCI treatment	0.60 (0.23–1.59)	0.307	161
In-hospital cardiac arrest	1.06 (0.50–2.23)	0.885	161
Witnessed arrest	0.36 (0.15–0.84)	0.018	161
Bystander CPR among OHCA	0.20 (0.04–1.00)	0.050	51
No-flow time, per minute	0.94 (0.74–1.19)	0.600	161
Recorded CPR duration, per minute	0.98 (0.93–1.03)	0.408	161
Low-flow time, per minute	1.06 (1.03–1.08)	<0.001	161
Initial shockable rhythm	0.41 (0.20–0.83)	0.013	161
Exact arrest/CPR timing	1.91 (0.82–4.46)	0.135	161
VAV ECMO mode	4.32 (0.54–34.49)	0.168	161
Recorded ECMO-startup interval, per minute	1.05 (1.03–1.08)	<0.001	161
APACHE II score, per point	1.06 (1.01–1.11)	0.015	161
Lactate, per mmol/L	1.12 (1.01–1.23)	0.031	161
LAD culprit among PCI recipients	0.77 (0.35–1.68)	0.512	130
TIMI grade 3 among PCI recipients	2.17 (0.83–5.70)	0.116	130

Univariable logistic regression with unfavorable neurological outcome (CPC 3-5) as the dependent variable. Bystander CPR was evaluated only among OHCA patients (*n* = 51). LAD culprit vessel and TIMI grade 3 were evaluated only among PCI recipients (*n* = 130). CI, confidence interval; CPC, cerebral performance category; CPR, cardiopulmonary resuscitation; ECMO, extracorporeal membrane oxygenation; LAD, left anterior descending; OHCA, out-of-hospital cardiac arrest; OR, odds ratio; PCI, percutaneous coronary intervention; STEMI, ST-segment elevation myocardial infarction; TIMI, thrombolysis in myocardial infarction; VAV, venoarterial-venous.

As shown in Table 4, prolonged low-flow time was associated with higher odds of CPC 3-5 after multivariable adjustment (aOR 1.065, 95% CI 1.036–1.096; *P* < 0.001 per minute) in 161 complete cases with 118 events. Prior coronary artery disease, unwitnessed arrest, and higher lactate also had *P* < 0.05. The apparent model C-statistic was 0.861, the Hosmer-Lemeshow *P* value was 0.742, and all variance-inflation factors were below 1.2. Across 1,000 bootstrap refits, all models converged, mean optimism was 0.035, and the optimism-corrected C-statistic was 0.826 (Supplementary Tables S5, S7).

**Table 4:** Primary multivariable logistic model with continuous low-flow time.

Variable	*β*	SE	aOR (95% CI)	*P* value
Age, per year	0.035	0.020	1.04 (1.00–1.08)	0.075
Male sex	0.891	0.492	2.44 (0.93–6.40)	0.070
Diabetes mellitus	−0.729	0.512	0.48 (0.18–1.32)	0.154
Coronary artery disease history	0.924	0.465	2.52 (1.01–6.27)	0.047
Witnessed arrest	−1.157	0.531	0.31 (0.11–0.89)	0.029
Low-flow time, per minute	0.063	0.014	1.07 (1.04–1.10)	<0.001
Lactate, per mmol/L	0.177	0.071	1.19 (1.04–1.37)	0.012
Initial shockable rhythm	−0.751	0.464	0.47 (0.19–1.17)	0.106

Multivariable logistic regression model adjusted for all variables shown, based on 161 complete cases (no missing data in any model variable). aOR, adjusted odds ratio; CI, confidence interval; SE, standard error.

Adding no-flow time, arrest location, PCI, APACHE II, STEMI, or timing precision one at a time produced only small changes in the continuous low-flow estimate (aOR range 1.063–1.067 per minute) (Supplementary Table S3). In a parsimonious model that included no-flow time, continuous low-flow time remained associated with unfavorable outcome (aOR 1.065, 95% CI 1.037–1.095; *P* < 0.001) (Supplementary Table S4). In the 132 patients with exact timing (100 unfavorable and 32 favorable outcomes), the low-flow association remained in both the primary-covariate model (aOR 1.082, 95% CI 1.042–1.124; *P* < 0.001) and the parsimonious model (aOR 1.079, 95% CI 1.043–1.117; *P* < 0.001). In the parsimoniously adjusted arrest-setting interaction model, the estimated aOR per minute was 1.061 (95% CI 1.027–1.096) for IHCA and 1.072 (95% CI 1.023–1.124) for OHCA; the interaction was not statistically detectable (*P* = 0.708) (Supplementary Table S6). These sensitivity analyses support a consistent direction of association but do not establish equivalent effects across settings; precision was limited in the smaller OHCA stratum.

### Dose-response and ROC analyses

The RCS knots were 24.8, 55.5, and 77.4 min. The estimated probability increased progressively across the observed low-flow range, with wider uncertainty at the extremes. The likelihood-ratio test did not detect departure from linearity (*P* = 0.660); thus, the analysis supports treating low-flow time continuously and provides no evidence of a biological inflection at 50.9 min or elsewhere (Figure 1).

**Figure 1:**
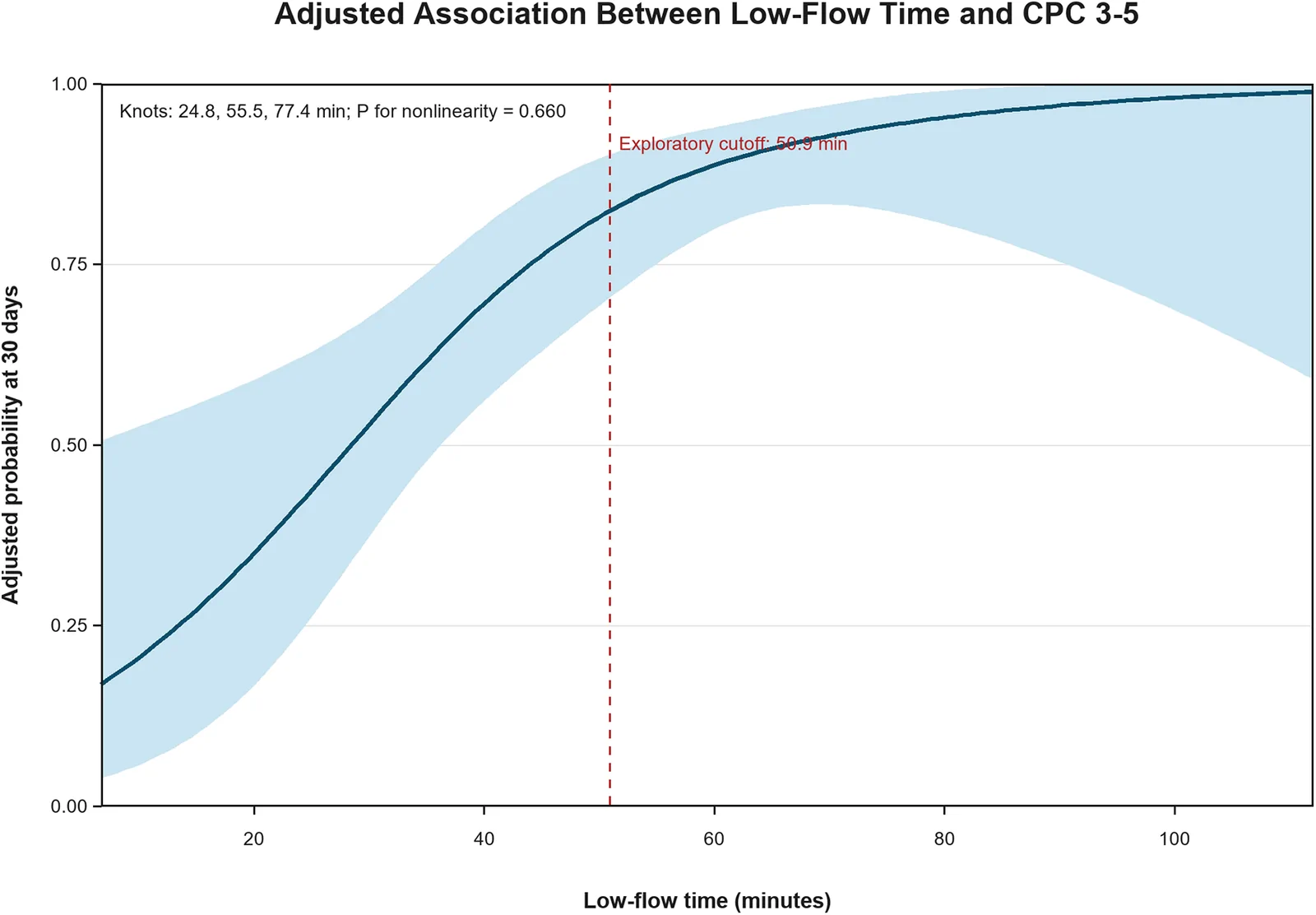
Adjusted dose-response relationship between low-flow time and unfavorable neurological outcome. The solid line shows the adjusted predicted probability of 30-day CPC 3-5, and the shaded area denotes the 95% confidence interval. The model was adjusted for age, sex, diabetes mellitus, coronary artery disease history, witnessed arrest, lactate, and initial shockable rhythm. The dashed line marks the exploratory 50.9-min cutpoint; it is not evidence of a biological inflection.

As a secondary exploratory analysis, low-flow time alone showed moderate discrimination (Figure 2; AUC 0.7634; bootstrap 95% CI 0.6759–0.8445). The maximum-Youden value was 50.85 min, but bootstrap-selected cutpoints were widely distributed (IQR 38.1–52.6; 95% interval 34.5–58.8 min). This instability indicates that 50.9 min is only a cohort-specific classification and not a biological inflection or validated clinical threshold.

**Figure 2:**
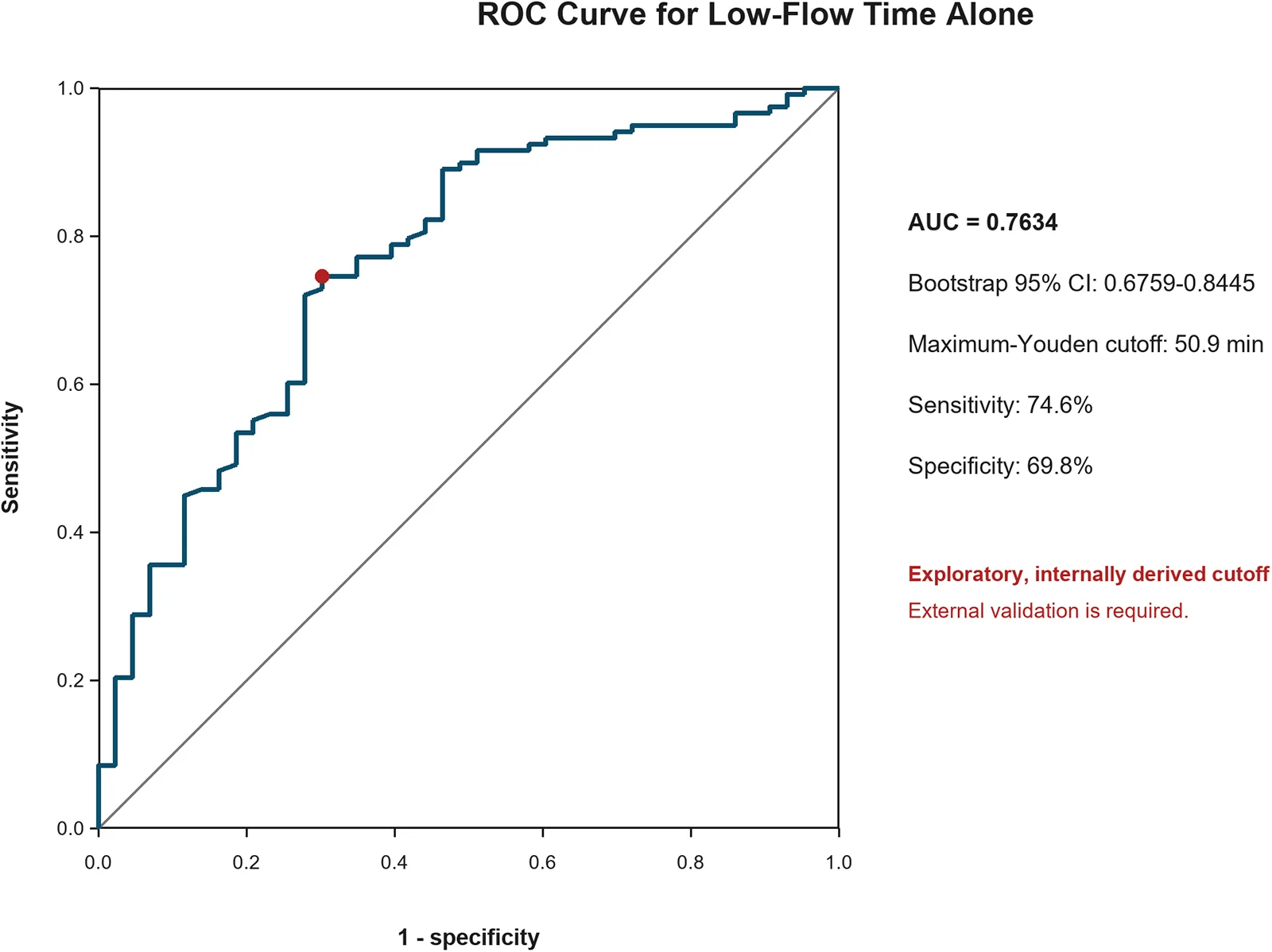
ROC curve for low-flow time as a predictor of unfavorable neurological outcome. This receiver operating characteristic (ROC) curve shows the discrimination of low-flow time alone for 30-day unfavorable neurological outcome in adult patients with AMI-related refractory cardiac arrest who received ECPR. The analysis is univariable and does not include covariate adjustment. The red marker denotes the internally selected maximum-Youden point.

In the exploratory dichotomized analysis, 30 of 60 patients below 50.9 min and 88 of 101 patients at or above 50.9 min had CPC 3-5. The adjusted aOR was 9.90 (95% CI 3.76–26.02; *P* < 0.001) (Table 5). Because the classification and its effect estimate were derived in the same cohort, and because the bootstrap cutpoint interval spanned 34.5–58.8 min, this large aOR should be interpreted as an unstable contrast between internally selected groups rather than as the effect of crossing a causal or clinical threshold. The continuous association is the principal finding.

**Table 5:** Exploratory multivariable model using low-flow time ≥50.9 min.

Variable	*β*	SE	aOR (95% CI)	*P* value
Age, per year	0.048	0.019	1.05 (1.01–1.09)	0.014
Male sex	0.899	0.493	2.46 (0.93–6.45)	0.068
Diabetes mellitus	−0.747	0.530	0.47 (0.17–1.34)	0.159
Coronary artery disease history	1.217	0.487	3.38 (1.30–8.77)	0.012
Witnessed arrest	−0.987	0.528	0.37 (0.13–1.05)	0.062
Low-flow time ≥50.9 min	2.292	0.493	9.90 (3.76–26.02)	<0.001
Lactate, per mmol/L	0.160	0.070	1.17 (1.02–1.35)	0.022
Initial shockable rhythm	−0.872	0.473	0.42 (0.17–1.06)	0.065

Exploratory multivariable logistic regression model with low-flow time dichotomized at the 50.9-min maximum-Youden cutpoint from ROC analysis, adjusted for all variables shown, based on 161 complete cases. aOR, adjusted odds ratio; CI, confidence interval; SE, standard error.

## Discussion

In this retrospective cohort of 161 AMI patients already selected for and treated with ECPR, the principal finding was a progressive association between longer low-flow time and unfavorable 30-day neurological outcome. Each additional minute was associated with a 6.5% increase in adjusted odds of CPC 3-5. Exact-timing and arrest-setting analyses showed a similar direction, whereas RCS provided no evidence of a nonlinear inflection. These are prognostic findings within an ECPR-treated cohort; they do not address whether ECPR should be initiated compared with alternative care.

Our findings are consistent with prior ECPR cohorts showing time-dependent associations (6–9), so the existence of a low-flow association is not itself novel. The incremental contribution is the focused AMI-CA context, in which coronary occlusion and reperfusion coexist with arrest-related ischemia; detailed coronary and timing-quality characterization; and direct evaluation of exact timing and IHCA/OHCA heterogeneity in a Chinese ECPR center. The absence of detectable interaction does not prove equivalence, especially with only 51 OHCA patients. Xu et al. reported an AUC of 0.750 and an exploratory cutoff of 55.5 min in prehospital ECPR (12), whereas the ELSO consensus lists arrest-to-ECMO flow under 60 min only as an example selection feature with acknowledged uncertainty (13). Together, these data support continuous prognostic information, not a universal exclusion rule.

A contribution of the present work is its focus on patients with cardiac arrest secondary to acute myocardial infarction (AMI). The available data permitted more detailed characterization of arrest and coronary variables. No-flow time was short in this cohort, in which 68.3% of arrests occurred in hospital; all IHCA values were ≤1 min and all cohort values were ≤5 min. The lack of a detectable no-flow association should not be interpreted as evidence of no effect because the range was restricted (11). Most patients underwent PCI, and TIMI grade 3 flow was documented in 83.8% of PCI recipients. Comparable symptom-to-PCI or arrest-to-reperfusion analyses could not be performed because symptom-onset time was unavailable and many coronary timestamps were internally discordant (10).

Additional registry studies, cohort analyses, and systematic reviews provide context for ECPR outcome assessment and cohort heterogeneity. Early withdrawal of life-sustaining therapy may influence outcome ascertainment (15). Initial or converted rhythm and sex have been associated with ECPR outcomes in observational studies (16, 17). Reviews emphasize arrest setting, patient selection, and ECPR-system organization (18, 19). Studies of arrest-to-ECMO duration and Chinese ECPR practice further support a time-sensitive, system-level interpretation rather than a universal cutoff (20, 21), and other refractory-arrest cohorts similarly reinforce the need for careful selection (22).

Current guidance recommends considering ECPR only for selected patients within systems able to deliver it promptly (23). Randomized trials have shown heterogeneous effects across different ECPR pathways and populations (4, 5, 24). Observational studies and a systematic review support interpreting low-flow time as a progressive time-outcome association; they do not establish a single biologically validated threshold (25–27). In cardiac-origin refractory arrest, programs combining rapid ECPR deployment with immediate invasive assessment and coronary treatment provide relevant system-level context (28).

From a clinical standpoint, the continuous relationship is more informative than the dichotomized contrast. RCS did not identify a biological inflection, and 50.9 min only summarizes discrimination within this cohort. The wide bootstrap interval makes the adjusted aOR of 9.90 sensitive to where patients are divided and likely exaggerates the apparent precision of a single cutpoint. Most importantly, every participant had already been selected for ECPR; therefore, neither the continuous association nor the exploratory classification estimates the benefit of initiating versus withholding ECPR. Low-flow time should be integrated with the wider clinical context rather than used as a stand-alone eligibility rule.

Post-ECPR temperature management was characterized at the protocol level. All patients were managed under a protocol aimed at normothermia and fever prevention, predominantly using surface methods; induced hypothermic temperature control at 32 °C–34 °C was not used. Because individual targets, device details, treatment durations, and rewarming rates were unavailable, temperature management could not be evaluated as a patient-level exposure (14, 29, 30).

Several limitations warrant consideration. First, this single-center retrospective cohort included only patients selected for ECPR, creating treatment-selection and survivor-process biases and precluding inference about ECPR eligibility or treatment benefit. Second, the smaller outcome group included 43 favorable outcomes for eight predictor parameters (5.4 per predictor). Although all 1,000 bootstrap fits converged, apparent C-statistic decreased from 0.861 to 0.826 after optimism correction, and the median test calibration slope was 0.792, indicating residual overfitting risk. Exact-timing and interaction analyses further reduced information, especially in OHCA, and should be interpreted as sensitivity analyses rather than definitive subgroup evidence. Third, follow-up was limited to 30 days, and the endpoint combined death with severe neurological disability. CPC assessor identity, blinding, and inter-rater reliability were unavailable. Agonal breathing, serial pupillary findings, reliable patient-level Impella/IABP use, standardized echocardiographic or Swan-Ganz time points, symptom-to-reperfusion intervals, and patient-level temperature-management details were unavailable. The primary model was not prespecified, and the RCS and cutpoint analyses were exploratory. Independent multicenter validation is required.

The study also has several methodological strengths. We employed multiple analytical approaches—logistic regression, RCS regression, and ROC analysis—to examine the relationship between low-flow time and outcome rather than relying on a single method. Additional strengths include explicit separation of no-flow and low-flow intervals, expanded AMI and arrest characterization, reporting of the complete CPC distribution, bootstrap assessment of model optimism and cutpoint stability, and transparent exclusion of non-comparable variables. These features improve transparency but do not eliminate residual confounding or support causal or threshold-based clinical claims.

## Conclusions

In this single-center Chinese cohort of AMI patients already treated with ECPR, increasing low-flow time was progressively associated with higher odds of unfavorable 30-day neurological outcome. The association remained in exact-timing records and did not show detectable heterogeneity between IHCA and OHCA, although subgroup precision was limited. No nonlinear inflection was detected. The findings are prognostic within selected ECPR recipients and do not establish an initiation, withholding, or eligibility threshold; the 50.9-min classification is exploratory and cohort-specific.

## Data Availability

The raw data supporting the conclusions of this article will be made available by the authors, without undue reservation.
