# Supplementary material for: Low-flow time and neurological outcomes in AMI patients undergoing ECPR: a retrospective cohort study
Source: Front Cardiovasc Med. 2026 Sep 14;13:1942551. doi: 10.3389/fcvm.2026.1942551 (PMC13617027; doi:10.3389/fcvm.2026.1942551)
Supplement: Supplementary file 1 [file Datasheet1.pdf]

Supplementary Methods

Source-data verification and analytic cohort

During post-review source-data verification, the previously assembled 167-record working cohort was reconciled against the available source documentation. Six records were excluded because serious contradictions in recorded event times prevented reliable determination of the primary low-flow interval (initiation of chest compressions to establishment of stable ECMO flow). The exclusions were based on timing-variable verifiability rather than neurological outcome. The final analytic cohort comprised 161 patients, all of whom had complete data for the primary low-flow exposure, the 30-day CPC endpoint, and the primary-model covariates.

No imputation was performed. Variables applicable only to a clinical subgroup were analyzed using their appropriate denominators: bystander CPR among patients with OHCA, culprit vessel and post-PCI TIMI flow among PCI recipients, and reasons for no PCI among patients who did not undergo PCI. Variables lacking a consistent time anchor or internally coherent patient-level timestamps were not repaired or inferred.

Statistical analyses

The primary multivariable logistic model evaluated low-flow time continuously and included age, sex, diabetes mellitus, prior coronary artery disease, witnessed arrest, lactate, and initial shockable rhythm. The smaller outcome group included 43 favorable outcomes (5.4 per predictor parameter). Newly retrieved variables were added one at a time. Exact-timing analyses repeated the primary model and a parsimonious five-predictor model. Arrest-setting heterogeneity was evaluated in the full cohort with a mean-centered low-flow-by-OHCA interaction adjusted for age, witnessed arrest, lactate, and initial shockable rhythm. Internal validation used 1,000 bootstrap samples.

A restricted cubic spline with knots at the 10th, 50th, and 90th percentiles assessed departure from linearity. Low-flow time alone was assessed by ROC analysis. The maximum-Youden midpoint was 50.85 minutes and was operationalized as <50.9 versus ≥50.9 minutes because low-flow time was recorded to one decimal place. The AUC and cutpoint distribution were evaluated in 2,000 bootstrap samples; optimism-corrected model C-statistics were estimated using 1,000 bootstrap samples. Statistical analyses were performed in R version 4.6.1, and final figures were prepared in GraphPad Prism 10.

Supplementary Results

The final cohort included 161 patients: 43 favorable and 118 unfavorable outcomes. Timing precision was exact in 132, estimated in 25, and indeterminate in 4 patients. Among exact records, low-flow time remained associated with unfavorable outcome in the primary-covariate model (aOR 1.082, 95% CI 1.042-1.124) and the parsimonious model (aOR 1.079, 95% CI 1.043-1.117). In the full-cohort, parsimoniously adjusted interaction model, the estimated slopes were 1.061 for IHCA and 1.072 for OHCA, with no statistically detectable interaction (P=0.708). This finding does not establish equivalence because precision was limited in the OHCA stratum. These analyses assess robustness within the treated cohort and do not establish causality or ECPR eligibility.

The RCS analysis did not detect departure from linearity (P=0.660). Low-flow time alone yielded an AUC of 0.7634 (bootstrap 95% CI 0.6759–0.8445). The maximum-Youden midpoint was 50.85 minutes, with 74.6% sensitivity and 69.8% specificity. Bootstrap-selected cutpoints were dispersed (median 50.85 minutes, IQR 38.1–52.6; 95% interval 34.5–58.8), supporting treatment of 50.9 minutes as an exploratory, internally derived classification rather than a validated clinical threshold.

Arrest-setting estimates are presented from a full-cohort interaction model rather than separate fully adjusted subgroup models, because the OHCA stratum contained only 51 patients and 14 favorable outcomes. This approach preserves the requested comparison while limiting subgroup overfitting.

Supplementary Tables

Supplementary Table S1. Operational definitions and analysis handling

Variable	Operational definition	Primary source/anchor	Analysis handling
AMI-related refractory cardiac arrest	Persistent absence of sustained return of spontaneous circulation despite conventional CPR and advanced life support in a patient with a contemporaneous cardiology diagnosis of STEMI or NSTEMI, prompting ECPR pathway activation.	Cardiology, resuscitation, and ECPR records	Required for cohort eligibility; a uniform minimum advanced-life-support duration could not be verified retrospectively.
Arrest location	IHCA if arrest occurred after hospital arrival; OHCA if arrest occurred before hospital arrival.	Arrest and emergency records	Analyzed for all 161 patients; added to the primary model in a one-at-a-time sensitivity analysis.
Witnessed arrest	Collapse or loss of signs of circulation directly observed by another person or clinical staff.	Resuscitation, nursing, prehospital, or family record	Analyzed as a binary variable in the primary model.
No-flow time	Interval from documented collapse/arrest to the first chest compression.	Arrest and CPR-start timestamps; exact or estimated timing classification	Complete for 161 patients; analyzed continuously in sensitivity models.
Low-flow time	Interval from initiation of chest compressions to establishment of stable ECMO flow.	CPR-start and stable-ECMO-flow timestamps	Primary exposure; evaluated continuously, with RCS, and using an exploratory ROC-derived classification.
Recorded CPR duration	Separately recorded resuscitation-duration field in the source record.	Resuscitation record	Reported descriptively; not substituted for low-flow time because the operational anchor differed.
Recorded ECMO-startup interval	Separately recorded interval associated with ECMO team activation/setup and initiation.	ECPR/ECMO record	Reported descriptively; not substituted for low-flow time because the operational anchor differed.
Neurological outcome	Favorable: CPC 1–2 at 30 days. Unfavorable: CPC 3–5 at 30 days. Death was coded as CPC 5.	Final 30-day clinical database	Primary endpoint; complete for 161 patients.
Coronary variables	Receipt of PCI for all patients; culprit vessel and post-PCI TIMI flow among PCI recipients; recorded reason among patients without PCI.	Angiography and PCI records	Analyzed using conditionally applicable denominators; inconsistent interval timestamps were not repaired or analyzed.
Temperature control	Center-level protocol aimed at normothermia and fever prevention; surface cooling predominated; induced hypothermic temperature control at 32°C–34°C was not used.	Institutional protocol and author verification	Described at protocol level only because patient-level targets, devices, durations, and rewarming rates

			were unavailable.

AMI, acute myocardial infarction; CPC, Cerebral Performance Category; CPR, cardiopulmonary resuscitation; ECMO, extracorporeal membrane oxygenation; ECPR, extracorporeal cardiopulmonary resuscitation; IHCA, in-hospital cardiac arrest; NSTEMI, non-ST-segment-elevation myocardial infarction; OHCA, out-of-hospital cardiac arrest; PCI, percutaneous coronary intervention; RCS, restricted cubic spline; ROC, receiver operating characteristic; STEMI, ST-segment-elevation myocardial infarction; TIMI, Thrombolysis in Myocardial Infarction.

Supplementary Table S2. Availability and denominator rules for variables requested during peer review

Variable/domain	Available denominator	Data status	Prespecified handling
Primary low-flow exposure, 30-day CPC, and primary-model covariates	161/161	Complete in the final analytic cohort	Primary and sensitivity analyses used complete-case data; no imputation was performed.
Arrest location, witnessed arrest, and no-flow time	161/161	Complete; timing classified as exact in 132, estimated in 25, and indeterminate in 4	Analyzed for the full cohort.
Bystander CPR	51/51	Applicable only to OHCA	Denominator restricted to the 51 OHCA patients.
PCI treatment	161/161	130 received PCI; 31 did not	PCI status available for the full cohort.
Culprit vessel and post-PCI TIMI flow	130/130	Applicable only to PCI recipients	Reported only among the 130 PCI recipients.
Reason for no PCI	31/31	Applicable only to patients not receiving PCI	Reported only among the 31 patients without PCI.
Angiography/reperfusion intervals	130 PCI records checked	68/130 records discordant with the arrest date; 62 were temporally plausible	All patient-level interval analyses were excluded because the source field was not consistently reliable; no corrections were inferred.
Symptom-onset time	Not uniformly available	Missing or not time-anchored across the cohort	Not analyzed.
Echocardiographic and Swan–Ganz variables	Not comparable	Echocardiography was obtained at nonuniform time points; Swan–Ganz data were incomplete and not time-anchored	Not analyzed because between-patient comparison would be misleading.
Temperature targets/devices/duration /rewarming	Protocol level only	Individual implementation details were not retained as analyzable fields	Described narratively; not entered into regression models.
Impella/IABP, pupillary findings, and agonal breathing	Not reliably available	No complete cohort-wide patient-level fields	Acknowledged as unavailable; not imputed or analyzed.

Denominators reflect logical applicability rather than missingness when a variable is defined only for OHCA, PCI, or non-PCI patients. IABP, intra-aortic balloon pump; OHCA, out-of-hospital cardiac arrest; PCI, percutaneous coronary intervention; TIMI, Thrombolysis in Myocardial Infarction.

Supplementary Table S3. Stability of the continuous low-flow estimate after adding newly retrieved covariates one at a time

Added covariate	N/events	Low-flow aOR per minute (95% CI)	P value	C-statistic / maximum VIF
No-flow time, per minute	161/118	1.066 (1.036–1.096)	<0.001	0.862 / 1.15
In-hospital cardiac arrest	161/118	1.065 (1.036–1.096)	<0.001	0.861 / 1.16
PCI treatment	161/118	1.065 (1.036–1.096)	<0.001	0.862 / 1.15

APACHE II score, per point	161/118	1.067 (1.033–1.101)	<0.001	0.862 / 1.53
STEMI	161/118	1.065 (1.035–1.096)	<0.001	0.860 / 1.15
Exact rather than estimated timing	161/118	1.063 (1.034–1.094)	<0.001	0.870 / 1.14

Each listed covariate was added separately to the same primary model (age, sex, diabetes mellitus, prior coronary artery disease, witnessed arrest, lactate, initial shockable rhythm, and continuous low-flow time). The added covariates were not entered simultaneously. aOR, adjusted odds ratio; APACHE II, Acute Physiology and Chronic Health Evaluation II; CI, confidence interval; PCI, percutaneous coronary intervention; STEMI, ST-segment-elevation myocardial infarction; VIF, variance inflation factor.

Supplementary Table S4. Parsimonious sensitivity model including no-flow time

Covariate	Adjusted OR	95% CI	P value
Age, per year	1.040	1.002–1.079	0.039
Witnessed arrest	0.357	0.134–0.952	0.040
No-flow time, per minute	0.861	0.653–1.137	0.292
Low-flow time, per minute	1.065	1.037–1.095	<0.001
Lactate, per mmol/L	1.199	1.049–1.369	0.008
Initial shockable rhythm	0.582	0.249–1.361	0.212

N=161; unfavorable-outcome events=118. Model C-statistic=0.840; Hosmer–Lemeshow P=0.620; maximum VIF=1.11. CI, confidence interval; OR, odds ratio; VIF, variance inflation factor.

Supplementary Table S5. Model performance, exploratory cutpoint, and internal validation

Metric	Continuous primary model	Exploratory threshold model	Low-flow-only ROC analysis
Exposure	Low-flow time, per minute	Low-flow <50.9 vs ≥50.9 minutes	Low-flow time alone
N / unfavorable events	161 / 118	161 / 118	161 / 118
Adjusted association	1.065 (1.036–1.096)	9.898 (3.765–26.021)	Not applicable
P value for association	<0.001	<0.001	Not applicable
Discrimination	C-statistic 0.861	C-statistic 0.861	AUC 0.7634 (bootstrap 95% CI 0.6759–0.8445)
Calibration	Hosmer–Lemeshow P=0.742	Hosmer–Lemeshow P=0.601	Not applicable
Optimism-corrected C-statistic	0.826	0.828	Not estimated
Maximum VIF	1.14	1.09	Not applicable
Exploratory cutpoint	Not applicable	50.85-minute midpoint, operationalized as 50.9 minutes	Sensitivity 74.6%; specificity 69.8%
Bootstrap cutpoint distribution	Not applicable	Not used to refit a new threshold	Median 50.85; IQR 38.1–52.6; 95% interval 34.5–58.8 minutes
RCS nonlinearity	P=0.660; knots 24.8, 55.5, and 77.4 minutes	Not applicable	Not applicable
Threshold-group event counts	Not applicable	<50.9: 30/60; ≥50.9: 88/101	Not applicable

The adjusted threshold-model effect compares ≥50.9 with <50.9 minutes. The 50.9-minute classification was derived and evaluated in the same cohort and should not be used as a stand-alone clinical decision threshold. AUC, area under the receiver operating characteristic curve; CI, confidence interval; RCS, restricted cubic spline; ROC, receiver operating characteristic; VIF, variance inflation factor.

Supplementary Table S6. Exact-timing and arrest-setting sensitivity analyses

Analysis	N / unfavorable events	Low-flow OR per minute (95% CI)	P value	Interaction P
Exact timing; primary-covariate model	132 / 100	1.082 (1.042-1.124)	<0.001	Not applicable
Exact timing; parsimonious model	132 / 100	1.079 (1.043-1.117)	<0.001	Not applicable
IHCA; unadjusted setting-specific slope	110 / 81	1.055 (1.026-1.085)	<0.001	0.631
OHCA; unadjusted setting-specific slope	51 / 37	1.068 (1.023-1.116)	0.003	0.631
IHCA; parsimoniously adjusted setting-specific slope	110 / 81	1.061 (1.027-1.096)	<0.001	0.708
OHCA; parsimoniously adjusted setting-specific slope	51 / 37	1.072 (1.023-1.124)	0.004	0.708

Exact timing means the precision field was classified as exact. The exact-timing primary-covariate model used the eight predictors in the main model; the parsimonious exact-timing model included age, witnessed arrest, continuous low-flow time, lactate, and initial shockable rhythm. Arrest-setting slopes were estimated from full-cohort interaction models; the parsimonious interaction model additionally adjusted for age, witnessed arrest, lactate, and initial shockable rhythm. Low-flow time was mean-centered before forming the interaction. Interaction P values compare the low-flow slopes for OHCA versus IHCA. These sensitivity analyses were not prespecified and do not establish equivalence.

Supplementary Table S7. Additional primary-model stability diagnostics

Diagnostic	Result	Interpretation
Smaller outcome group	43 favorable outcomes	5.4 patients per predictor parameter for eight predictors
Bootstrap convergence	1000/1000 fits	All resamples converged
Apparent C-statistic	0.861	Performance in the development cohort
Mean bootstrap optimism	0.035	Difference between bootstrap and original-sample performance
Optimism-corrected C-statistic	0.826	Lower than apparent performance
Median test calibration slope	0.792	Below 1, consistent with residual overfitting
Bootstrap low-flow OR	median 1.071; 2.5th-97.5th percentiles 1.040-1.111	Positive in all 1,000 bootstrap samples

The bootstrap used 1,000 resamples of 161 patients with refitting of the complete primary model. The calibration-slope distribution was broad (2.5th-97.5th percentiles 0.517-1.136), so these internal estimates do not replace external validation.
